# Reconsidering tools for measuring gender dimensions in biomedical research

**DOI:** 10.1186/s13293-024-00663-7

**Published:** 2024-11-25

**Authors:** Rosemary Morgan, Anna Yin, Anna Kalbarczyk, Janna R. Shapiro, Patrick J. Shea, Helen Kuo, Carmen H. Rodriguez, Erica N. Rosser, Andrew Pekosz, Sean X. Leng, Sabra L. Klein

**Affiliations:** 1 Department of International Health, Johns Hopkins Bloomberg School of Public Health, Baltimore, MD USA; 2 W. Harry Feinstone Department of Molecular Microbiology and Immunology, Johns Hopkins Bloomberg School of Public Health, Baltimore, MD USA; 3 Department of Emergency Medicine, Johns Hopkins University, Baltimore, MD USA; 4 Department of Medicine, Division of Geriatric Medicine and Gerontology, Johns Hopkins Center on Aging and Immune Remodeling, Johns Hopkins University, Baltimore, MD USA

**Keywords:** Gender role measurement tools, Gender scores, Gender scales, Gender-based analyses

## Abstract

Sex and gender play important roles in contributing to disease and health outcomes and represent essential, but often overlooked, measures in biomedical research. The context-specific, multifaceted, and relational nature of gender norms, roles, and relations (i.e., gender dimensions) make their incorporation into biomedical research challenging. Gender scores—measures of gender dimensions—can help researchers incorporate gender into quantitative methodologies. These measures enable researchers to quantify the gendered dimensions of interest using data collected from survey respondents. To highlight the complexities of using gender scores within biomedical research, we used the application of the Bem Sex Role Inventory (BSRI) scale, a commonly used gender score, to explore gender differences in adverse events to the influenza vaccine among older adults (75+). Within this paper, we focus on the findings from our longitudinal gender score data collected over three influenza seasons (2019-20, 2020-21, and 2021-22), irrespective of adverse event data, to provide commentary on the reliability of gender scores, such as the BSRI, and the complexities of their application. Of the 162 total study participants included within the study, 69 were enrolled in all three consecutive seasons and 35 participants were enrolled in two consecutive seasons. The majority of participants had a different gender score in at least one of the years, demonstrating the nuances and fluidity of gender identity. Interpretations of BSRI data (or other gender score data) when measured against outcome data must, therefore, be time and context specific, as results are unlikely to be replicated across years.

## Highlights

Gender impacts health behaviors and outcomes but can be difficult to capture with a quantitative tool due to its complex nature.

Gender scores enable researchers to quantify gendered dimensions (e.g., gender norms, roles, and relations) using data collected from surveys.

Due to the fluidity of gender, established gender scores may no longer be appropriate nor effective. Gender scores will, therefore, need to be continuously adapted and/or created to fit the population and time of interest.

Biomedical researchers should not shy away from using gender scores within their research. They should carefully consider how gender data is collected, using relevant and appropriate gender scores for their area of interest, and interpreting their results accordingly.

## Introduction

Sex and gender play important roles in contributing to disease and health outcomes and represent essential, but often overlooked, measures in biomedical research. Here, sex is defined as the biological characteristics (e.g., sex chromosomes, sex steroid hormones, anatomy, etc.) that define humans as female, male, or intersex [1]. Gender is a social construct relating to the norms and roles associated with being a man, woman, or an identity beyond these categories, and relations between groups [2]. The biological differences between males and females impact one’s risk of disease, manifestation of illness, immune responses to treatment, and many other aspects of one’s health and well-being [1]. On the other hand, gender norms, roles, and relations impact access to important health resources, health behaviors, and health outcomes. Gender dimensions include: access to resources, such as knowledge, education, or financial resources; decision-making around who gets to seek care and when; practices that can increase risk of poor health, such as substance abuse, smoking, and violence; norms around what is appropriate for men and women and gender-minority individuals; and care-seeking behaviors [3]. Gender is, therefore, an important social determinant of health.

Within biomedical research, gender is often conceptualized as gender identity—whether a person identifies as being a woman, man, or gender minority individual. How this is captured in research also differs, for example, by directly asking whether someone is a woman, man, or gender minority individual. There are different ways to ask about a person’s gender identity due to the variations in genders that exist. The National Academies of Sciences, Engineering, and Medicine have issued guidance for researchers on how to measure sex, gender identity, and sexual orientation [4], which can be used to navigate these complexities. Gender norms, roles, and relations are different from, but related to, gender identity in that they are more systemic, simultaneously influencing how society is organized more broadly in terms of social norms, institutions, structures, resources, interpersonal relationships between individuals (particularly men and women), and a person’s individual gender identity. Asking about a person’s gender identity can help disaggregate data and explore differences between groups but will not get at the ways in which gender norms, roles, and relations impact a person’s access to important health resources, health behaviors, and health outcomes.

The context-specific, multifaceted, and relational nature of gender norms, roles, and relations (hereafter referred to as gender dimensions) makes its incorporation into biomedical research challenging. Many aspects of gender can also be difficult to quantify, making its incorporation into research methodologies, such as surveys or questionnaires, even more difficult. Gender scores—measures of gender dimensions—can help researchers incorporate gender into quantitative methodologies, which are commonly used in biomedical research. These measures enable researchers to calculate the presence of the gendered phenomena of interest using data collected from survey respondents.

While not exhaustive, the gender scores that have been utilized in biomedical research include: the Bem Sex Role Inventory (BSRI) scale [5–7], the Gender and Sex Determinants of Cardiovascular Disease: From Bench to Beyond-Premature Acute Coronary Syndrome questionnaire (GENESIS-PRAXY), which embeds the BSRI in its items [8, 9], the Conformity to Masculine Norms Inventory (CMNI) [10], the Gender Role Conflict Scale (GRCS) [11], the Personal Attributes Questionnaire (PAQ) [12], and the Gender Equitable Men’s Scale (GEMS) [13]. Many of these scores seek to assess participants’ identification with traditional masculine and feminine traits, which capture different gender dimensions as a proxy for gender. Specifically, trait measures recognize that an individual may possess both masculine and feminine characteristics and these may represent the extent of an individual’s adherence to cultural gender norms [14]. Other measures, such as the GEMS and CMNI, measure gender ideologies by assessing an individual’s endorsement of a culture’s ideological beliefs about gender roles. Select scores are further described in Table 1. These gender scores have been used across an array of medical fields, including obesity [5], cancer [11, 15], mental health [6, 16], injuries [17, 18], and heart disease [8, 19, 20], with mixed results.

**Table 1:** Select gender scores used in biomedical research

Gender score	What is measured	Strengths	Limitations
**Trait Measures**			
Bem Sex Role Inventory (BSRI)	Constructs of masculinity; femininity; androgyny; undifferentiation. Several adaptations of the original BSRI are in use today.	Validated in different populations (in older people) Short survey	Uses outdated stereotypes.
Personal Attributes Questionnaire (PAQ)	Constructs of masculinity and femininity rated as “ideal” and then the extent to which they apply to participants.	Validated in different populations including children.	Older, using outdated ideations of gender.
**Ideology Measures**			
Conformity to Masculine Norms Inventory (CMNI)	The degree to which participants adhere to norms of masculine ideologies: winning, emotional control, risk-taking, violence, dominance, playboy, self-reliance, primacy of work, power over women, distain for homosexuality, and pursuit of status.	Expanded the scope of masculine norms.	Lengthy tool
Gender Role Conflict Scale (GRCS)	Men’s responses to situations where masculine gender role expectations are challenged or unrealistic. The following subscales are measured: success, power and competition (SPC); restricted emotionality (RE), restricted affectionate behavior between men (RABBM); conflict between work and family relationships.	Widely used among various US ethnic groups and men from other countries. Robust psychometric properties.	Only three of the subscales directly measure restrictive gender roles that cause gender conflict.

For example, one study conducted in 2007 using the short form of the BSRI reported that men with higher femininity scores had a lower risk of coronary heart disease, yet the same relationship was not observed among women [21]. Another study using the GENESIS-PRAXY gender score found worse cardiovascular health and a higher prevalence of heart disease were associated with gender roles and personality traits typically ascribed to women, regardless of the individual’s sex, among Canadian and Austrian population samples [20]. In both populations, gender correlated more strongly with a higher risk of heart disease than sex. Another study using the BSRI examined the role of gender in treatment adherence among participants with bipolar disorder and found that males with high masculinity scores were nearly four times more likely to not adhere to medication when compared to males who did not have high masculinity characteristics [16]. No significant relationship between gender scores and adherence was found in females. While gender is closely tied to an individual’s health-seeking behaviors, access to healthcare, and gender-related health risks, the consideration of gender in biomedical studies remains limited [22].

To highlight the complexities of using gender scores within biomedical research, we explored the application of the BSRI, a commonly used gender score, to evaluate sex and gender differences in immunological response and adverse events to the influenza vaccine among older adults (75+) [23–26]. A description of the study, its methodology, and BSRI findings are provided below.

### Application of the BSRI

In this paper, we focus on the findings from our longitudinal gender score data, irrespective of adverse event data, to provide commentary on the reliability of gender scores, such as the BSRI, and the complexities of their application. The BSRI was originally developed in 1974 and used a 12-item scale of masculine traits (e.g., leadership abilities, strong personality, acts as a leader, dominant, makes decisions easily, and defends own beliefs) and feminine traits (e.g., warmth, gentleness, affection, sympathy, sensitivity to others’ needs, and tenderness), which were representative of the time to ascertain whether a person was feminine, masculine, androgynous, or undifferentiated [7, 23].

We applied the BSRI within a larger vaccination study, the Johns Hopkins Longitudinal Influenza Immunization Study of Aging (JH-LIISA), which recruited participants during the 2019-20, 2020-21, and 2021-22 influenza seasons in Baltimore, Maryland, United States. We used a modified short form of the 12-item BSRI to calculate femininity and masculinity scores and to assign participants to one of the four gender categories. A five-point Likert scale, ranging from 1 = never to 5 = always, was used to measure each trait and totaled, with gender categories assigned relative to the sample median feminine and masculine score as previously described [7, 23]. Androgyny combines masculine and feminine traits, and undifferentiated describes people whose scores on feminine and masculine traits were low [7]. The BSRI was chosen for this study as it has been used and validated in populations of older adults in multiple different cultures over the past decade [7, 23, 27–29]. Despite (or perhaps the result of) being developed over five decades ago [30], it is one of the most commonly used and validated measures of gender dimensions [7].

**Fig. 1:**
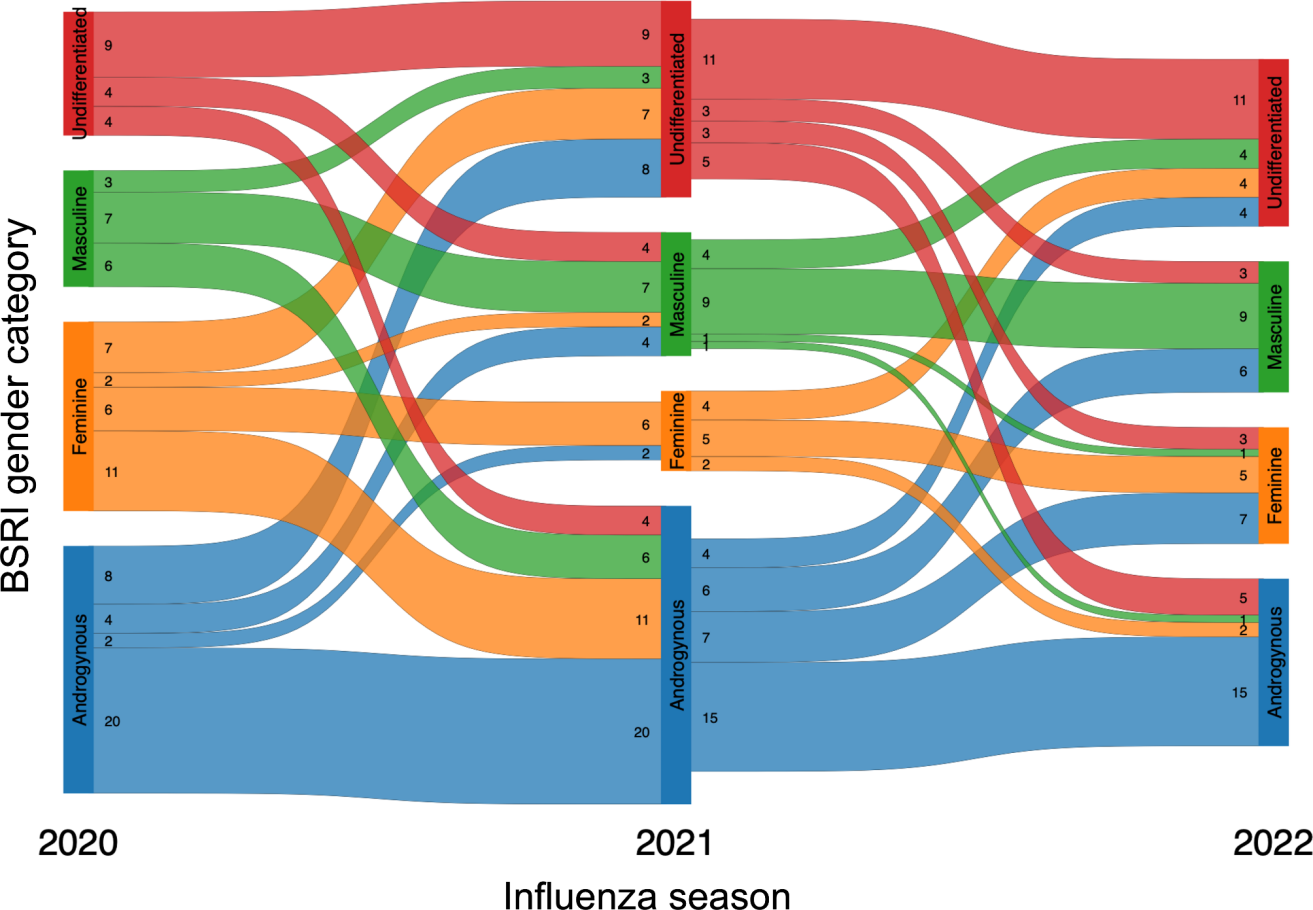
Graphic representation of gender categories based on participants’ scoring via the Bem-Sex Role Inventory (BSRI), connected and color-coded by gender category across two or three seasons, depending on participant enrollment, of a longitudinal influenza vaccine study. The chords show participant BSRI scores from year-to-year. Chords connecting from the same color between years represent participants with unchanging BSRI scores. Chords connecting different colors between years represent changes in gender scoring. Number of participants within each category is noted. (*n* = 162; all three seasons *n* = 69; two consecutive seasons *n* = 35)

As previously published, the odds of reporting an adverse event following influenza vaccination did not depend on the gender category, but rather biological sex, among older adults (75+) [23]. Here, we explored whether participants’ gender scores remained the same or differed from year-to-year to better understand the reliability of gender scores over time. Of the 162 total study participants included within JH-LIISA, 69 were enrolled in all three consecutive seasons and 35 participants were enrolled in two consecutive seasons. 32% of the 69 participants (*n* = 22 total) enrolled in all three consecutive seasons scored within the same BSRI gender category from year-to-year, while 46% of the 35 participants (*n* = 16) enrolled in two consecutive seasons scored within the same BSRI gender category from in both years (Fig. 1). Most participants changed in how their gender was categorized by the BSRI (*n* = 47 of 69, 68% of those enrolled in all three seasons; *n* = 19 of 35, 54% of those enrolled in two consecutive seasons). Pearson correlation coefficients were calculated for the BSRI masculinity and femininity scores for consecutive years (2019–2020 and 2020–2021) to assess retest reliability, with r values ranging from 0.52 to 0.58 (all *p* < 0.001), suggesting moderate reliability of the BSRI from year-to-year. These changes demonstrate the nuances and fluidity of gender identity and how interpretations of BSRI data, when measured against outcome data, must be time and context-specific as results are unlikely to be replicated across years.

## Discussion

Differences in health outcomes between and among women, men, and gender minority individuals cannot all be equated to biological sex differences. There is clear evidence of the role that gender norms, roles, and relations play in shaping health outcomes [1–3, 31]. It is, therefore, essential that gender is taken into consideration within biomedical research. The inclusion of gender scores is one way in which biomedical researchers have attempted to account for gender, and many biomedical studies which have included gender score data have demonstrated a relationship between gender and health outcomes [5, 6, 15, 16, 19–21, 32].

Gender scores currently utilized within biomedical research are diverse, using different methodologies to study gender and its role on health outcomes. The diversity of gender scores and the measures used reflect the multifaceted and context-specific nature of gender norms, roles, and relations. Gender dimensions manifest through men and women’s differential access to resources, roles and practices, norms and beliefs, decision-making power and autonomy, as well as institutions, laws, and policies [33, 34]. Understanding the impact of gender dimensions on health outcomes requires a multidimensional approach; it is not enough to simply ask questions about access to resources, roles, or norms on their own—multiple questions spanning the different dimensions are needed. How you select which questions to include will depend on the topic being studied and its context. As it is impractical to include all gender dimensions, some gender dimensions will need to be prioritized over others. Gender frameworks [33, 34] and gender analysis matrices [35] can be utilized to help identify and prioritize relevant gender dimensions.

While there are many similarities between men and women, what it means to be a man or woman (and what it means to not fit within gender binaries) differs based on one’s racial/ethnic culture, political and/or religious climates, location, and time. Traditional notions of masculinity and femininity (as depicted in the BSRI) are grounded in gendered stereotypes, which in a developed, secular country like the U.S. can become quickly outdated. Donnelly and Twenge [36] explored how masculine and feminine traits on the BSRI changed on college campuses in the U.S. from 1993 to 2012 through a meta-analysis of cross-sectional surveys. They found that women’s femininity scores decreased significantly between 1993 and 2012, whereas their masculinity scores remained the same. No significant changes were found for men. An expanded analysis comparing data from 1974 (when the BSRI first came out) to 2012 found that women’s masculinity scores rose significantly over this time. Overall, their findings showed that women in U.S. colleges have become less likely to endorse typical “feminine” traits as representative of themselves. The authors concluded, however, that it is possible the scale items no longer match modern conceptions of gender and gender dimensions, and that future research may need to update the BSRI. When compared to modern day notions of gender, however, it becomes clear that the stereotypical masculine and feminine gender traits contained in the BSRI no longer apply.

It is worth mentioning that our study was conducted among older adults (75+). These adults have likely witnessed a substantial shift in gender dimensions over time, from when the BSRI was developed until now. Women’s movements throughout the 20th century challenged traditional gender norms and roles, advocating for increased women’s participation within the workplace, reproductive freedom, and a greater role for men within the home and as caregivers. While gendered stereotypes persist (and detrimentally affect women, men, and gender minority individuals), people are likely to see many of the traits categorized within the BSRI as not being specific to being masculine or feminine. Over time, it has become more acceptable for men, women, and gender minority individuals to embody non-traditional gender traits. The change that Donnelly and Twenge [36] found in their study among college students is likely a reflection of this shift. Evidence of the BSRI among older adults [7] shows that for men and women, gender identity is less rigid, which reflects a complex shifting of gender norms and roles rather than a dichotomous construct. It is also possible that as women and men age, gender norms, roles, and relations become less stringent (or less important), and individuals are more able to embody diverse traits with little to no repercussions.

Many of our participants’ gender scores changed from year-to-year, which could reflect how individuals responded to questions about themselves and how they saw themselves at the time of survey administration. This is also likely a reflection of how gender dimensions change—not only across decades, but also from year-to-year. We know that gender inequities affect health behaviors and outcomes, and as such, it is important to try to understand the role of different gender dimensions beyond trait measures related to gender. However, due to the context and time specific nature of gender, the type of gender score and how it is applied become important. Many gender score data may only ever be able to be understood cross-sectionally as a reflection of a specific time and place, and as such, results need to be interpreted accordingly. That is, the context in which the data is collected needs to be considered when designing future studies, interventions, or policies to address the role of gender in health. Based on the limitations of the scores identified in this review, as well as the findings from our application of the BSRI, we have generated recommendations to guide the development of a gender score and analysis of gender score data (Table 2).

**Table 2:** Recommendations for developing a gender score and analyzing gender score data

• Ensure that the gender dimensions and questions included are context-specific and relevant to the topic being studied.
• Use a multidimensional approach—ask questions across multiple gender dimensions (e.g., access to resources, roles and practices, norms and beliefs, decision-making power and autonomy) while prioritizing dimensions and questions that are most relevant for the topic and context.
• Ensure that the gender score includes gender dimensions beyond trait measures related to gender (e.g., concepts of masculinity and femininity).
• Regularly update the gender score to reflect current norms, ensuring that the gender score does not perpetuate harmful gender stereotypes.
• Analyze and interpret the gender score data as a reflection of a specific time and place.
• Analyze data across relevant social stratifiers, such as race, ethnicity, age, education, disability, income, etc.

Overall, evidence shows that gender scores can be useful in understanding the impact of gender norms, roles, and relations on health behaviors and outcomes. Due to the multifaceted and multidimensional nature of gender, however, it can be difficult to capture in a quantitative tool. Due to the shifting nature of gender, it is important that researchers ensure that gender scores like the BSRI, which include outdated views of masculinity and femininity, are updated to reflect a more modern understanding of gender. Gender scores, therefore, will likely need to be continuously adapted and/or created to fit the topic and time of interest. Researchers should not shy away from using gender scores within their biomedical research; instead, they should carefully think about how data on gender is collected, using relevant and appropriate gender scores for their area of interest, and interpreting their results accordingly.

## Data Availability

No datasets were generated or analysed during the current study.

## Abbreviations

BSRI: Bem Sex Role Inventory
GENESIS-BRAXY: Gender and Sex Determinants of Cardiovascular Disease: From Bench to Beyond-Premature Acute Coronary Syndrome questionnaire
CMNI: Conformity to Masculine Norms Inventory
GRCS: Gender Role Conflict Scale
GEMS: Gender Equitable Men’s Scale
PAQ: Personal Attributes Questionnaire
JH-LIISA: Johns Hopkins Longitudinal Influenza Immunization Study of Aging
